# The Vaginal Microbiota, Human Papillomavirus Infection, and Cervical Carcinogenesis: A Systematic Review in the Latina Population

**DOI:** 10.1007/s44197-024-00201-z

**Published:** 2024-02-26

**Authors:** Vianney Mancilla, Nicole R. Jimenez, Naomi S. Bishop, Melissa Flores, Melissa M. Herbst-Kralovetz

**Affiliations:** 1 Department of Basic Medical Sciences, University of Arizona College of Medicine-Phoenix, 425 N. 5th Street, Phoenix, AZ 85004-2157 USA; 2 Department of Obstetrics and Gynecology, University of Arizona College of Medicine-Phoenix, 425 N. 5th Street, Phoenix, AZ 85004-2157 USA; 3 Associate Librarian, University of Arizona Health Sciences, University of Arizona College of Medicine-Phoenix, 475 N. 5th Street, Phoenix, AZ 85004 USA; 4 Department of Psychology, University of Arizona, 1200 E University Boulevard, Tucson, AZ 85721 USA

**Keywords:** Microbiome, Cervical cancer, Health disparities, Human papillomavirus, Latin America and the Caribbean, Latinas

## Abstract

**Background:**

Latina women experience disproportionately higher rates of HPV infection, persistence, and progression to cervical dysplasia and cancer compared to other racial–ethnic groups. This systematic review explores the relationship between the cervicovaginal microbiome and human papillomavirus infection, cervical dysplasia, and cervical cancer in Latinas.

**Methods:**

The review abides by the Preferred Reporting Items for Systematic Reviews and Meta-analyses guidelines. PubMed, EMBASE, and Scopus databases were searched from January 2000 through November 11, 2022. The review included observational studies reporting on the cervicovaginal microbiota in premenopausal Latina women with human papillomavirus infection, cervical dysplasia, and cervical cancer.

**Results:**

Twenty-five articles were eligible for final inclusion (*N* = 131,183). Forty-two unique bacteria were reported in the cervicovaginal microbiome of Latinas. Seven bacteria: *Lactobacillus crispatus, Lactobacillus iners, Chlamydia trachomatis, Prevotella* spp.,* Prevotella amnii, Fusobacterium* spp. and *Sneathia* spp. were enriched across multiple stages of cervical carcinogenesis in Latinas. Therefore, the total number of reported bacteria includes four bacteria associated with the healthy state, 16 bacteria enriched in human papillomavirus outcomes, 24 unique bacteria associated with abnormal cytology/dysplasia, and five bacteria associated with cervical cancer. Furthermore, three studies reported significantly higher alpha and beta diversity in Latinas with cervical dysplasia and cancer compared to controls. *Lactobacillus* depletion and an increased abundance of *L. iners* in Latinas compared to non-Latinas, regardless of human papillomavirus status or lesions, were observed.

**Conclusions:**

The identification of 42 unique bacteria and their enrichment in cervical carcinogenesis can guide future cervicovaginal microbiome research to better inform cervical cancer prevention strategies in Latinas.

**Supplementary Information:**

The online version contains supplementary material available at 10.1007/s44197-024-00201-z.

## Introduction

Cervical cancer is the fourth most common cancer affecting women worldwide [1]. Incident rates vary among high- and low-income countries due to public health efforts targeting this preventable disease. As a result of population-based cancer screening and human papillomavirus (HPV) vaccination programs, cervical cancer incidence and mortality rates have largely declined in high-income countries [1]. However, in Latin America and the Caribbean (LAC), HPV prevalence and cervical cancer mortality are among the highest in the world [2]. By 2025, 126,000 Latinas are predicted to be diagnosed with cervical cancer in LAC—a 75% rise in the frequency of the disease from 2002 [2].

Latina women are disproportionately affected by risk factors for HPV infection, persistence, and progression to cervical dysplasia and cancer [3–9]. Latinas experience the highest rates of HPV infection, with an incidence of over 40%, and are 40% more likely to be diagnosed with cervical cancer compared to other racial–ethnic groups [6–8]. In addition, Latinas have the highest cervical cancer incidence of any racial–ethnic group and are 24% more likely to die from cervical cancer compared to non-Hispanic White women [7]. This disparity could be exacerbated by systemic barriers that prevent Latinas from receiving adequate healthcare services, including HPV vaccination, cervical cancer screenings, and health education [2, 8, 9].

The premalignant precursor of cervical cancer, cervical intraepithelial neoplasia (CIN), is caused by persistent infection with high-risk HPV genotypes [10]. Although over 90% of HPV infections are cleared, reinfection of HPV can occur, and persistent HPV infection is linked to carcinogenesis [11]. The optimal environment that promotes HPV clearance is not completely understood; however, evidence shows that cervicovaginal microbiota play a dual role in HPV clearance or persistence and cervical cancer development and progression [10, 12, 13].

A cervicovaginal microbiome dominated by *Lactobacillus* species is often a proxy for vaginal health. *Lactobacillus* species facilitate homeostasis by creating a lactic acid-enriched microenvironment; this competitive niche adaptation indirectly protects the host from invading pathogens [10, 14–16]. Bacterial vaginosis (BV) is characterized by a depletion in *Lactobacillus* species and an increase in microaerophilic and anaerobic microbes, such as *Fannyhessea*/*Atopobium, Gardnerella, Prevotella, and Sneathia* species [15–18]. Notably, BV is associated with an increased risk of sexually transmitted infections (STIs), including HPV [10, 14–21].

Two recent systematic reviews in non-Hispanic White women have (1) established a causal link between vaginal dysbiosis and cervical carcinogenesis [12] and (2) assessed community state types in women with HPV infection, cervical dysplasia, and cervical cancer [22]. However, an individual analysis of specific cervicovaginal bacteria in Latina women who are particularly high risk for adverse gynecologic sequelae has not been conducted. The cervicovaginal microbiota of Latinas must be further investigated, given the significantly high rates of vaginal dysbiosis, HPV infection, and cervical cancer in this population [2, 3, 6–8, 21, 23, 24].

## Objectives

The purpose of the present review was to identify bacteria reported in the cervicovaginal microbiome of  Latinas relating to HPV infection, cervical dysplasia, and cervical cancer as well as better understand the role of the microbiome in these disease states worldwide. These data could provide novel insights and approaches to address health disparities in HPV infection, persistence, and cervical cancer morbidity and mortality in this historically understudied, underrepresented, and underreported population of women.

## Methods

This systematic review followed the PRISMA (Preferred Reporting Items for Systematic Review and Meta-Analyses) guidelines and was registered in PROSPERO (CRD42022367244) on 11/21/2022 [25]. No similar systematic review or protocol was registered.

### Eligibility Criteria, Information Sources, Search Strategy

Studies in which participants were pre-menopausal women identifying as Hispanic or Latina according to the U.S Census Bureau definition: “a person of Cuban, Mexican, Puerto Rican, South or Central American, or other Spanish culture or origin regardless of race” were included in the systematic review [26]. Furthermore, studies were only included if participants were diagnosed with either HPV, dysplasia, and/or cervical cancer or were healthy controls for studying these disease states. In addition, studies were required to be observational and describe analyses related to cervicovaginal microbiota. Interventional studies, article reviews, or commentaries were excluded. Studies evaluating pregnant people or non-human subjects were also excluded.

The following electronic databases were searched from January 2000 through November 11, 2022: PubMed, EMBASE (Elsevier), and Scopus. A search of ClinicalTrials.gov was not applicable because the studies for this review were not interventional. The search strategy employed a combination of terms related to “vagina/cervix” and “microbiota.” This review used the **Latinx/Hispanic US Population Search Hedge** developed by the Medical Library Association Latinx Caucus for this literature search [27]. The search was restricted to articles published after January 2000 on “female(s)” with no language restrictions. A more detailed report of the search strategy can be found in Supplementary Table 1. All records were exported into the bibliographic software EndNote X9 to remove duplicates. The unique records were then transferred to a web-based systematic review software, DistillerSR [28].

### Study Selection

Titles and abstracts of retrieved articles were screened independently by two authors (V.M., N.R.J.). Any discrepancies were included for full-text screening to determine eligibility. The same two authors (V.M., N.R.J.) independently screened full-text articles for eligibility, with discrepancies resolved by a third author (M.M.H–K).

### Data Extraction

Two authors (V.M., N.R.J.) independently performed data extraction. A standardized data collection form in DistillerSR was created to collect: (1) participant characteristics (i.e., age, menopausal status, and gender identity), (2) geographic characteristics, (3) study characteristics (i.e., recruitment site, sample collection method, number of participants) (4) clinical characteristics (i.e., HPV genotypes, indication of abnormal Papanicolaou (Pap) smears, histological stage/grade, and comorbidities), and (5) microbiome methodology (i.e., identification methods for the microbiome and HPV, community state types, *Lactobacillus* dominance/depletion, alpha and beta diversity, and bacteria associated with outcome groups). Discrepancies in data collection were resolved after discussion and reviewing the full-text article. The compiled data from the full-text extraction are provided in Supplementary Table 2.

### Assessment of Risk of Bias

The Risk of Bias in Non-randomized Studies of Exposure (ROBINS-E) tool was utilized to evaluate the risk of bias for observational studies [29]. The ROBINS-E tool evaluates each study according to seven domains of bias: (1) due to confounding, (2) arising from measurement of the exposure, (3) in selection of participants into the study (or into the analysis), (4) due to post-exposure interventions, (5) due to missing data, (6) arising from measurement of the outcome, and (7) in selection of the reported result. The risk of bias for each domain and overall bias for each study was consolidated into a summary table; see Table 1.

**Table 1:** ROBINS-E Risk of Bias for Included Articles

ID#	First Author	Domain 1: Risk of bias due to confounding^a^	Domain 2: Risk of bias arising from measurement of the exposure	Domain 3: Risk of bias in selection of participants into the study	Domain 4: Risk of bias due to post-exposure interventions	Domain 5: Risk of bias due to missing data	Domain 6: Risk of bias arising from measurement of the outcome	Domain 7: Risk of bias in selection of the reported result	Overall risk of bias
1	Nieves-Ramirez et al.	Low	Low	Some concerns	Low	High	Some concerns	Some concerns	High
2	Mosmann et al.	Some concerns	Low	Some concerns	Some concerns	Low	Low	Some concerns	Some concerns
3	Carrillo-Ng et al.	High	Low	Some concerns	Some concerns	Some concerns	Low	Low	High
4	Hernandez-Rosas et al.	High	Low	Some concerns	Some concerns	Some concerns	Low	Low	High
5	Conde-Ferráez et al.	Very High	⎽	⎽	⎽	⎽	⎽	⎽	Very High
6	Vargas-Robles et al.	High	Low	Some concerns	Some concerns	Some concerns	Low	Low	High
7	Torres-Poveda et al.	High	Low	Some concerns	Some concerns	Low	Low	Low	High
8	Bristow et al.	Very High	⎽	⎽	⎽	⎽	⎽	⎽	Very High
9	Romero-Morelos et al.	High	Low	Some concerns	Some concerns	Some concerns	Low	Low	High
10	Melo et al.	Very High	⎽	⎽	⎽	⎽	⎽	⎽	Very High
11	Mongelos et al.	Some concerns	Low	Some concerns	Some concerns	Low	Low	Low	Some concerns
12	Lippman et al.	High	Low	Some concerns	Some concerns	Some concerns	Some concerns	Low	High
13	Soto et al.	Very High	⎽	⎽	⎽	⎽	⎽	⎽	Very High
14	DeLuca et al.	Very High	⎽	⎽	⎽	⎽	⎽	⎽	Very High
15	Tonon et al.	High	Low	Some concerns	Some concerns	High	Low	Low	High
16	Somesh-Vikramdeo et al.	Very High	⎽	⎽	⎽	⎽	⎽	⎽	Very High
17	Manzanares-Leal et al.	High	Low	Some concerns	Some concerns	Some concerns	Low	Low	High
18	Sanchez-Garcia et al.	High	Low	Some concerns	Some concerns	Low	Low	Low	High
19	de Oliveira Ignacio et al.	Very High	⎽	⎽	⎽	⎽	⎽	⎽	Very High
20	Godoy-Vitorino et al.	High	Low	Some concerns	Some concerns	Some concerns	Low	Low	High
21	Łaniewski et al.	Some concerns	Low	Some concerns	Some concerns	Some concerns	Low	Low	Some concerns
22	Gomes de Oliveira et al.	High	Low	Some concerns	Some concerns	High	Low	Low	High
23	Audirac-Chalifour et al.	Low	Low	Some concerns	Some concerns	Low	Low	Low	Some concerns
24	Clarke et al.	High	Low	Some concerns	Some concerns	Some concerns	Low	Low	High
25	Escarcega-Tame et al.	High	Low	Some concerns	Some concerns	High	Low	Low	High

ROBINS-E risk of bias associated with each of the seven domains and overall risk of bias for each included study are reported. The level of bias can be categorized as low risk, some concerns, high risk, or very high risk. The dash symbol denotes “no rating.” The ROBINS-E assessment concludes as very high risk after reporting the highest risk of bias rating in Domain 1. ^a^Five confounding variables must be controlled to rate Domain 1 as low risk

### Data Synthesis

A data collection form was developed and completed for each article in DistillerSR. Data was synthesized in a tabulated form by DistillerSR and downloaded as an Excel spreadsheet (Supplementary Table 2).

## Results

### Study Selection

**Fig. 1:**
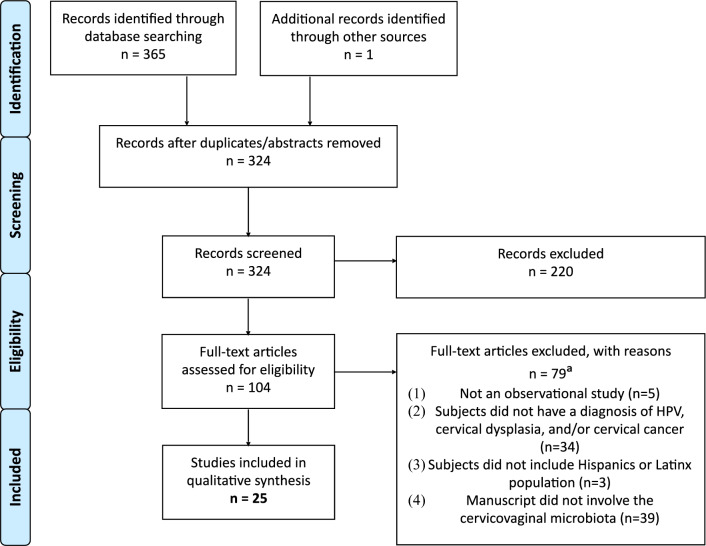
PRISMA Methodological Flowchart. The flowchart depicts the identification, selection, and final inclusion of articles. Records of excluded articles and reasons for exclusion are also included. ^a^The total number of full-text articles excluded was *n* = 79. However, articles could be excluded for more than one reason. Two articles were excluded for multiple reasons, therefore the total sum for each reason is 81

The initial search yielded a total of 365 articles from three databases: PubMed (178), EMBASE (168), and Scopus (19). One article was included due to a similar and relevant title during the full-text upload of documents. Twenty-five conference abstracts and 17 duplicates were removed, resulting in 324 unique articles. A title–abstract screening was performed on 324 articles, leading to the exclusion of 220 articles. One hundred four full-text articles were assessed for eligibility. Seventy-nine studies were excluded for not meeting the inclusion criteria. Nearly, half of the excluded articles (39/79) were ineligible because they lacked information on the cervicovaginal microbiome. This search method yielded a final sample of 25 full-text articles; see Fig. 1 for the PRISMA methodological flowchart.

### Study Characteristics

The key characteristics of the 25 included observational studies are featured in Table 2. Some longitudinal studies were included but most studies were cross-sectional. The types of cross-sectional studies featured include descriptive, population-based, and prevalence studies. No qualitative studies were included. The studies were published between 2004 and 2022. The geographical regions of the studies encompass North [ID# 1, 4, 5, 7–9, 16–18, 20, 21, 23, 25], Central [ID# 24], and South America [ID# 2, 3, 6, 10–12, 14, 15, 19, 22, 24], including the Caribbean [ID# 13]. Studies from ten different countries were included. The total number of participants in the review was 131,183. With the exception of three articles [ID# 9, 13, 21], most studies reported the age of participants with the minimum reported age of 12 years old [ID# 6] and the maximum reported age of 100 years old [ID# 24]. The two most common methods of sample collection were by vaginal swab (*n* = 10, 40%) [ID# 1, 6, 8, 12, 17–21, 24] and cytobrush (*n* = 10, 40%) [ID# 2–5, 9–11, 14, 15, 19], followed by biopsy (*n* = 2, 8%) [ID# 7, 16], cervicovaginal lavage (*n* = 1, 4%) [ID# 19], endo/ectocervical scrapings (*n* = 1, 4%) [ID# 13], and endocervical swab (*n* = 1, 4%) [ID# 25]. Two studies did not report sample collection methods (*n* = 2, 8%) [ID# 22, 23]. In addition, two studies used multiple methods of sample collection, which accounts for the overall percentage above 100% [ID# 19, 21]. Of the 25 included articles, 22 studies reported on HPV outcomes (88%) [ID# 1, 3–7, 9–16, 18–25]. The most common HPV genotypes reported were HPV16, HPV31, and HPV6 (Supplementary Table 2). In addition, 13 studies [ID# 1, 2, 8, 9, 13–16, 20–23, 25] reported on abnormal cytology/dysplasia outcomes (46%), and four studies [ID# 17, 21, 23, 25] reported on cervical cancer outcomes (16%). Ten articles [ID# 1, 9, 13, 15, 16, 20–23, 25] reported on more than one outcome; three studies reported on all three outcomes [ID# 21, 23, 25].

**Table 2:** Study Characteristics and Key Findings of the Included Articles

ID#	First Author (Publication Year)	NParticipants	NOutcomeGrp	NCtrlGrp	Age range	Location	HPV outcome	Abnormal cytology outcome	Cervical cancer outcome	Sample collections	Bacteria identified	Other findings
1	Nieves-Ramirez et al. (2021)	228	121	107	21 +	Mexico City, Mexico	✓	✓	⎽	Vaginal swab	⎽	Alpha diversity: individuals with SILs had significantlty higher species richness and evenness compared to non-SILs; beta diversity: SILs explains 1.4% of the variation in vaginal bacterial community structure
2	Mosmann et al. (2021)	100	50	50	18–67	Córdoba, Argentina	⎽	✓	⎽	Cytobrush	*Chlamydia trachomatis*	⎽
3	Carrillo-Ng et al. (2021)	833	200	633	27–43	Cajamarca, Peru	✓	⎽	⎽	Cytobrush	*Eubacterium, Actinobacteria, Fusobacterium, Bacteroides, Bifidobacterium, Enterococcus*	⎽
4	Hernandez-Rosas et al. (2021)	1000	272	728	18–65	Sonora, Mexico	✓	⎽	⎽	Cytobrush	*Actinomyces*	⎽
5	Conde-Ferráez et al. (2017)	233	63	170	15–49	Merida, Yucatan, Mexico	✓	⎽	⎽	Cytobrush	*Chlamydia trachomatis*	⎽
6	Vargas-Robles et al. (2020)	111	74	21	12–53	Venezuela	✓	⎽	⎽	Vaginal swab	*Anaerococcus tetradius*, *Coriobacteriaceae*, *Prevotella* spp., *Prevotella amnii*	*Lactobacillus* dominance: *L. iners* was the most abundant taxa; alpha diversity: no associations were detected for any of the HPV types; beta diversity: microbiota diversity did not differ significantly according to HPV status
7	Torres-Poveda et al. (2019)	115,651	15,040	100,611	25–64	Mexico	✓	⎽	⎽	Biopsy	⎽	⎽
8	Bristow et al. (2019)	300	35	265	18 +	Tijuana, Mexico	⎽	✓	⎽	Vaginal swab	*Chlamydia trachomatis*	⎽
9	Romero-Morelos et al. (2018)	177	73	104	⎽	Guerrero, Mexico	✓	✓	⎽	Cytobrush	⎽	⎽
10	Melo et al. (2016)	151	57	94	18–24	Temuco, Chile	✓	⎽	⎽	Cytobrush	*Chlamydia trachomatis*	⎽
11	Mongelos et al. (2015)	181	42	139	23–41	Paraguay	✓	⎽	⎽	Cytobrush	*Chlamydia trachomatis, Mycoplasma hominis*	⎽
12	Lippman et al. (2010)	386	135	251	18–40	Sao Paulo, Brazil	✓	⎽	⎽	Vaginal swab	⎽	⎽
13	Soto et al. (2007)	60	30	30	⎽	Havana, Cuba	✓	✓	⎽	Ecto/endocervical scrapings	*Mycoplasma* spp.	⎽
14	DeLuca et al. (2006)	189	89	100	15–58	Resistencia, Argentina	✓	✓	⎽	Cytobrush	*Chlamydia trachomatis*	⎽
15	Tonon et al. (2004)	207	133	74	12–64	Misiones, Argentina	✓	✓	⎽	Cytobrush	*Gardnerella vaginalis*	⎽
16	Somesh-Vikramdeo et al. (2022)	11	7	4	20–62	Mobile, Alabama	✓	✓	⎽	Biopsy	*Rubellimicrobium, Pedobacter, Brevibacterium, Paracoccus, Fannyhessea, Brevundimonous, Comamonas, Novosphingobium*	*Lactobacillus* depletion: decreased abundance of *Lactobacillus* in Hispanic compared to Non-Hispanic White suggests an increased risk of colonization of the pathogenic microbes; alpha diversity: no difference in species richness and evenness according to CIN or HPV status
17	Manzanares-Leal et al. (2022)	120	60	60	21–71	Mexico	⎽	⎽	✓	Vaginal swab	*Corynebacterium amycolatum, Staphylococcus epidermidis*	⎽
18	Sanchez-Garcia et al. (2019)	201	78	123	18–50	Tabasco, Mexico	✓	⎽	⎽	Vaginal swab	*Chlamydia trachomatis*	⎽
19	de Oliveira Ignacio et al. (2018)	150	68	82	19–50 +	Botucatu, São Paulo, Brazil	✓	⎽	⎽	Vaginal swab, Cytobrush	⎽	⎽
20	Godoy-Vitorino et al. (2018)	62	52	10	21–50	San Juan, Puerto Rico	✓	✓	⎽	Vaginal swab	*Lactobacillus kitasatonis, Tissierella praeacuta, Lactobacillus acidophilus, Gardnerella vaginalis, Prevotella timonensis, Lactobacillus fornicalis, Prevotella amnii, Parvimonas micra, Lactobacillus crispatus*	*Lactobacillus* dominance: *L. iners* was the most common high-abundance bacterial taxon, present in more than 83% of the samples, regardless of HPV risk and lesions; alpha—Shannon bacterial diversity was significantly higher in introitus and cervix of CIN 3 compared to CIN 1 patients (P = 0.033 and P = 0.031)
21	Łaniewski et al. (2018)	100	49	51	⎽	Phoenix, Arizona	✓	✓	✓	Vaginal swab, Cervicovaginal lavage	*Lactobacillus iners, Sneathia* spp., *Fannyhessea* spp., *Parvimonas* spp., *Gardnerella vaginalis, Prevotella* spp., *Megasphaera* spp., *Lactobacillus crispatus, Shuttleworthia* spp.	*Lactobacillus* depletion: Hispanic ethnicity was associated with depletion of lactobacilli
22	Gomes de Oliveira et al. (2017)	1346	1192	154	17–81	Fortaleza, Brazil	✓	✓	⎽	Not specified	⎽	⎽
23	Audirac-Chalifour et al. (2016)	32	12	20	22–61	Morelos, Mexico & Mexico City, Mexico	✓	✓	✓	Not specified	*Sneathia* spp., *Megasphaera elsdenii, Shuttleworthia satelles, Lactobacillus iners, Lacobacillus crispatus, Fusobacterium necrophorum*	Beta diversity: the CC samples showed the highest variation among groups (HPV, cervical dysplasia, and CC); phylogenetic diversity—significant difference in SIL vs. CC where microbiota diversity in CC cases is higher than in the non-cervical lesions group
24	Clarke et al. (2012)	9165	3065	6100	18–100	Guanacaste, Costa Rica	✓	⎽	⎽	Vaginal swab	⎽	⎽
25	Escarcega-Tame et al. (2020)	189	107	82	18–50	Mexico City, Mexico	✓	✓	✓	Endocervical brush	*Chlamydia trachomatis*	⎽

The data extraction table reports study authors and publication year, number of participants, number of participants in the outcome and control group, the age range of participants, the location of the study, the types of outcomes studied: HPV, abnormal cytology or dysplasia, and cervical cancer, sample collection method, bacteria identified in outcome or control groups, and other microbiome metrics, such as *Lactobacillus* dominance, depletion, and diversity

### Quality Assessment

The quality of the included studies was assessed by performing the ROBINS-E risk of bias for each article (Table 1). Out of the seven domains assessed, the domain with the highest risk of bias was considered the study’s overall risk of bias [29]. The first domain, bias due to confounding, had the highest reported biases. For this domain, the authors developed a list of major risk factors influencing cervicovaginal microbiota composition and referred to confounding variables included in a similar systematic review during this process [12]. As a result, five major confounding factors influencing cervicovaginal microbiota composition were included: age [30, 31], parity [32], hormonal contraceptive use [33, 34], smoking [35], and STIs, including human immunodeficiency virus (HIV) [36, 37], herpes simplex virus (HSV) [38, 39], chlamydia [40, 41], gonorrhea [42, 43], and trichomoniasis [44, 45]. Seven articles did not control or adjust for the listed confounding variables contributing to their overall “very high” risk of bias score (Table 1) [ID# 5, 8, 10, 13, 14, 16, 19]. Thirteen articles controlled for a limited number of confounding variables contributing to their overall “high risk” of bias rating [ID# 3, 4, 6, 7, 9, 12, 15, 17, 18, 20, 22, 24, 25]. Three articles controlled for most confounding variables and were categorized as having “some concerns” of bias [ID# 2, 11, 21]. Two studies controlled for all five confounding variables and had a low risk of bias for Domain 1 [ID# 1, 23]. Due to further bias ratings, the two studies were categorized as having “some concerns” [ID# 23] and a “high” overall risk of bias [ID# 1]. Domains 2–7 were consistently rated as “some concerns” or “low” bias, with the exception of Domain 5 which resulted in four “high” bias ratings due to missing data [ID# 1, 15, 22, 25].

### Synthesis of Results

Eighteen studies reported on bacteria associated with outcome groups (Table 2) [ID# 2–6, 8, 10, 11, 13–18, 20, 21, 23, 25]. Five studies [ID# 1, 6, 16, 20, 23] reported on alpha diversity using the Chao index [ID# 1], Simpson’s index [ID# 6], Shannon index [ID# 6, 16, 20], observed features [ID# 16], and phylogenetic diversity  whole tree (Table 2 and Supplementary Table 1) [ID# 23]. In two studies, a significantly higher species richness and diversity were observed in Latinas with high-grade cervical intraepithelial neoplasia (CIN3) compared to low-grade cervical intraepithelial neoplasia (CIN1) [ID# 20] and Latinas with squamous intraepithelial lesions (SILs) compared to individuals without dysplasia [ID# 1]. One study reported on phylogenetic diversity and found that in Latinas, the microbiota diversity was higher in cervical cancer cases compared to the non-cervical lesions group [ID# 23]. Three studies reported on beta diversity using the Bray–Curtis distance metric [ID# 1], the Unweighted UniFrac distance metric [ID# 6, 23], and the Mann–Whitney *U* test (Table 2 and Supplementary Table 1) [ID# 23]. Regarding beta diversity, one study reported that 1.4% of the microbiome composition is contributed by SILs [ID# 1]. A second study reporting on beta diversity revealed that cervical cancer samples demonstrated the most variation in microbiota composition compared to HPV and cervical dysplasia groups [ID# 23]. Four studies reported on *Lactobacillus* [ID# 6, 16, 20, 21]. Both studies reporting on *Lactobacillus* dominance found that *Lactobacillus iners* was the most abundant bacteria amongst Latinas (*n* = 37, 45%; *n* = 48, 83%) [ID# 6, 20]. Moreover, two included studies revealed significant depletion of *Lactobacillus* species amongst Latinas compared to non-Latinas regardless of HPV status or lesions (*n* = 47, 42–86% [ID# 21]) [ID# 16, 21]. Statistical analyses performed in the aforementioned articles are listed in Table 2.

**Fig. 2:**
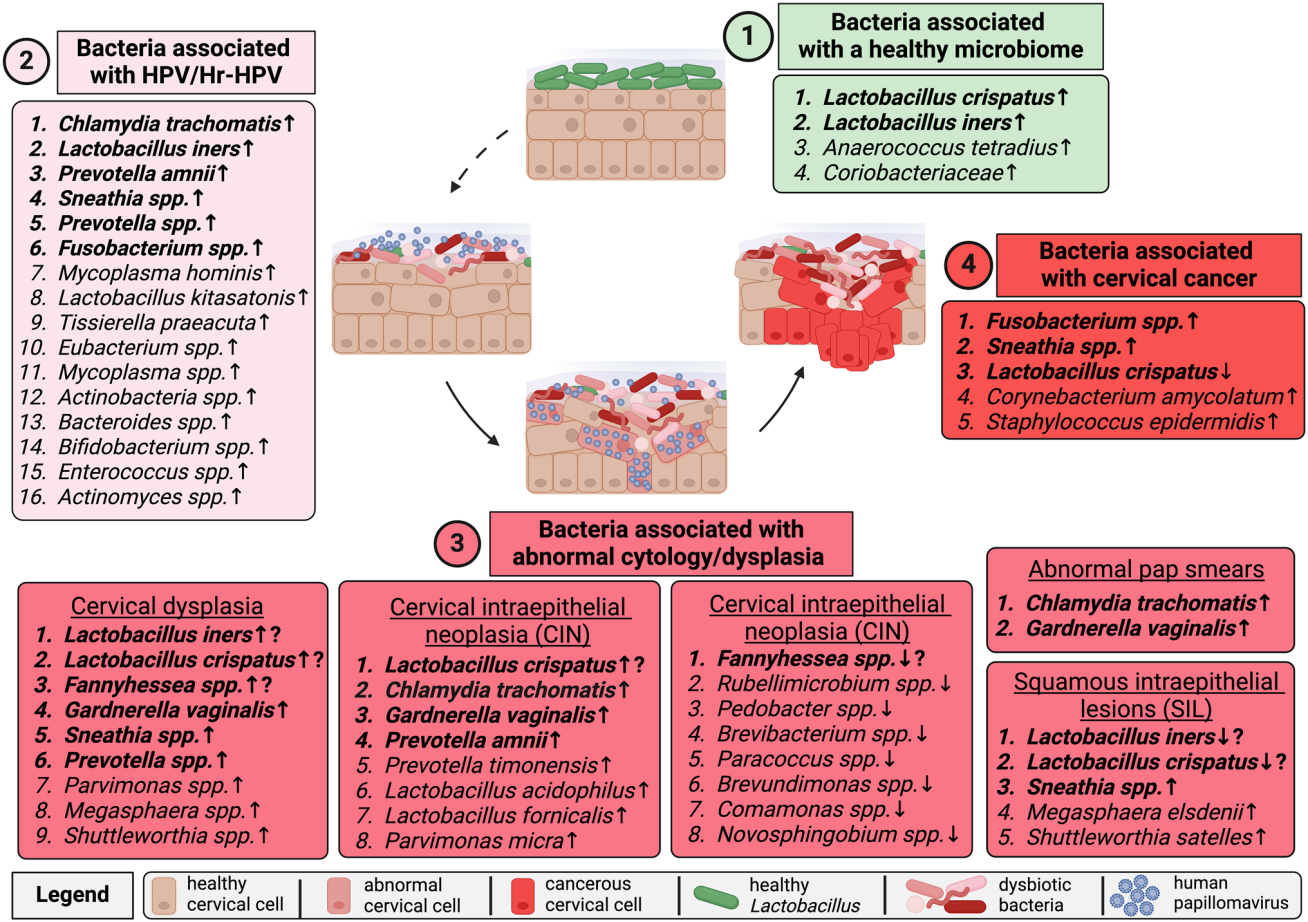
Cervicovaginal Bacteria Associated With Cervical Carcinogenesis in Latinas. Differences and similarities in the cervicovaginal microbiome composition from (1) healthy state to (2) HPV infection, (3) abnormal cytology/dysplasia, and (4) cervical cancer are depicted. Panel 1 is a light green color indicating an association with vaginal health. The following disease conditions proceed in a pink-to-red gradient according to severity: Panel 2 is light pink for HPV infection, Panel 3 is dark pink for abnormal cytology/dysplasia, and Panel 4 is bright red for cervical cancer. Enrichment or depletion of bacterial taxa associated with each stage is indicated with an up or down arrow, respectively. A question mark denotes differences in reports with regard to bacterial enrichment or depletion within the same panel. Bolded bacteria were reported in more than one study

The reported bacteria were categorized into each outcome group to display cervical cancer progression in Latinas; see Fig. 2. Panel 1 depicts a healthy microbiome in Latinas, which was associated with the enrichment of four bacteria: *Lactobacillus crispatus* [ID# 23], *L. iners* [ID# 23], *Anaeroccoccus* [ID# 6], and *Coriobacteriaceae* [ID# 6]. Panel 2 highlights 16 bacteria from nine articles indicating enrichment in Latinas that were HPV-positive or high-risk HPV (Hr-HPV) positive [ID# 3, 4, 6, 10, 11, 13, 14, 20, 21]. *Chlamydia trachomatis* was the most consistently reported bacteria in Latinas with HPV or Hr-HPV infection (*n* = 4, 1.7%, *n* = 17, 11.2%; *n* = 12, 21%; *n* = 100, 52.9%, *n* = 51, 28%) [ID# 5, 10, 11, 14, 25]. Panel 3 incorporates various conditions related to abnormal cytology or dysplasia, such as abnormal Pap smears, squamous intraepithelial lesions (SILs), cervical dysplasia, and cervical intraepithelial neoplasia (CIN), and highlights 32 total associations among 24 unique bacteria across nine articles [ID# 2, 8, 15, 16, 18, 20, 21, 23, 25]*. C. trachomatis* (*n* = 30, 60%; *n* = 17, 48.6%; *n* = 6, 3%) [ID# 2, 8, 18] and *Gardnerella vaginalis* (*n* = 42, 20%) [ID# 15] were enriched in Latinas with abnormal Pap smears. *Sneathia *spp., *Megasphaera elsdenii,* and *Shuttleworthia satelles* were enriched in Latinas with SILs, while *L. iners* and *L. crispatus* were depleted [ID# 23]. Nine bacteria were enriched in Latinas with cervical dysplasia [ID# 21]. In Latinas with CIN, eight bacteria were enriched [ID# 20]. One study reported on the depletion of eight bacteria in the vaginal microbiome of Latinas with CIN [ID# 16]. Overall, the following bacteria were consistently enriched in Latinas with abnormal cytology/dysplasia: *Sneathia *spp. (*n* = 3, 10.3% [ID# 23]) [ID# 21, 23], *C. trachomatis* (*n* = 30, 60% *n* = 17, 48.6%; *n* = 6, 3%, *n* = 11, 14.3%) [ID# 2, 8, 18, 25]*,* and *G. vaginalis* (*n* = 42, 20% [ID# 15]) [ID# 15, 20, 21]. Lastly*,* Panel 4 shows that Latinas with cervical cancer had enrichment of four cervicovaginal bacteria [ID# 17, 21, 23] and depletion of *L. crispatus* [ID# 23].

**Fig. 3:**
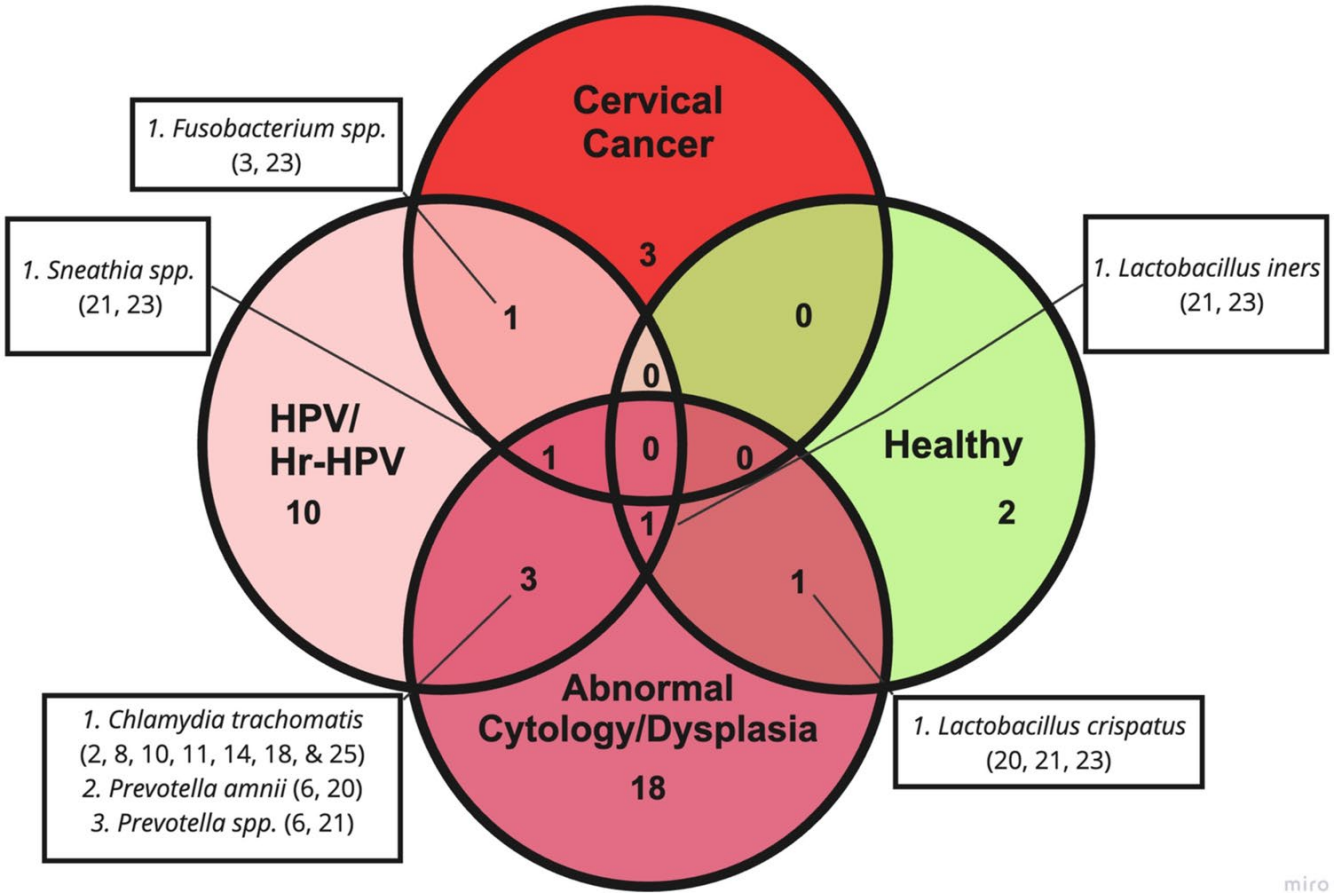
Cervicovaginal Bacteria Enriched Across Stages of Cervical Carcinogenesis in Latinas. The quadruple Venn diagram highlights seven bacteria enriched across outcome groups, including healthy controls. *Sneathia *spp. and *L. iners* were associated with multiple outcome groups across cervical carcinogenesis. The numbers listed after each bacterial species correspond to the article reporting on the enrichment of the bacteria (reference Table 2)

Approximately 42 unique bacteria in Latinas were associated with a healthy microbiome, HPV infection, abnormal cytology/dysplasia, and/or cervical cancer outcome groups. Some bacteria were enriched across multiple stages of cervical carcinogenesis in Latinas, which is summarized in a Venn diagram; see Fig. 3. *L*. *crispatus* was associated with health and abnormal cytology/dysplasia [ID# 20, 21, 23], whereas *L. iners* was associated with health, HPV/Hr-HPV infection, and abnormal cytology/dysplasia [ID# 21, 23]. Three bacteria, *C. trachomatis* (*n* = 30, 60%; *n* = 17, 48.6%; *n* = 17, 11.2%; *n* = 12, 21%; *n* = 100, 52.9%; *n* = 6, 3%; *n* = 51, 28%) [ID# 2, 8, 10, 11, 14, 18, 25], *Prevotella amnii* [ID# 6, 20], and *Prevotella *spp. [ID# 6, 21] were enriched in the HPV/Hr-HPV and abnormal cytology/dysplasia groups. Over a quarter of included studies reported *C. trachomatis* enrichment in HPV infection and abnormal cytology/dysplasia (*n* = 30, 60%; *n* = 17, 48.6%; *n* = 17, 11.2%; *n* = 12, 21%; *n* = 100, 52.9%; *n* = 6, 3%; *n* = 51, 28%) [ID# 2, 8, 10, 11, 14, 18, 25]. *Fusobacterium* and *Sneathia *spp. [ID# 21, 23] were enriched in Latinas with cervical cancer. *Sneathia *spp. were enriched in Latinas across all stages of cervical carcinogenesis [ID# 21, 23]. Specific statistical analyses in the aforementioned studies can be found in Supplementary Table 2.

## Discussion

### Principal Findings

This systematic review summarizes the currently available literature reporting on cervicovaginal bacteria associated with HPV infection, cervical dysplasia, and cervical cancer in North, Central, and South American Latinas. Of 324 studies, 25 articles met our inclusion criteria, revealing 42 unique cervicovaginal bacteria associated with HPV infection, cervical dysplasia, and/or cervical cancer in Latinas. Other significant findings related to alpha and beta diversity and *Lactobacillus* dominance or depletion in Latinas were also reported.

### Comparison with Existing Literature

In our review, two bacteria, *L. crispatus* and *L. iners,* were identified in two articles reporting on the healthy cervicovaginal microbiome of Latinas. The literature suggests that *L. crispatus* is associated with HPV-resistance and clearance which was confirmed in our review on Latinas [46]. *L. iners*, on the other hand, is not associated with HPV clearance and was consistently reported in women with bacterial vaginosis in a longitudinal study [46, 47]. In addition, our review revealed that in some studies, *L. crispatus* was reportedly enriched in health and abnormal cytology/dysplasia, while *L. iners* was enriched in health, HPV/Hr-HPV infection, and abnormal cytology/dysplasia in Latinas. These findings are supported by a systematic review of 29 articles which reports a generally high relative abundance of *L. iners* and a lower relative abundance of *L. crispatus* in non-Latina individuals with precancerous lesions and cervical cancer [48]. Although, the review did not define specific values for “high” and “low” abundances of *Lactobacillus* species. Multiple studies have linked *L. iners* enrichment to HPV infection [22, 49, 50] and even cervical cancer [22, 51]. In contrast to women with *L. iners*-dominant microbiomes, those with *L. crispatus*-dominant microbiomes have a more stable microenvironment and are more likely to clear HPV infection [13, 22, 52].

Sixteen bacteria were enriched in the cervicovaginal microbiome of Latinas with HPV/Hr-HPV infection across nine studies in our review. Across studies related to HPV/Hr-HPV infection, *C. trachomatis* and *P. amnii* were significantly enriched while four bacteria were consistently enriched, although not significant: *Sneathia* spp., *L. iners*, *Prevotella* spp., and *Fusobacterium *spp.. Among the listed bacteria, *C. trachomatis, P. amnii, and Prevotella *spp. were enriched across eight studies in our review reporting on Latinas with HPV/Hr-HPV infection and abnormal cytology/dysplasia. *C. trachomatis* infection has been previously associated with HPV and Hr-HPV infection and persistence [53]. In addition, HPV/*C. trachomatis* co-infection has been identified as a significant risk factor for cervical cytological abnormalities [54–57]. Notably, a study based in Morocco revealed that women coinfected with HPV/*C. trachomatis* were three times more at risk of developing cervical abnormalities [57]. Previous studies in non-Latina cohorts reveal an increased abundance of *Prevotella *spp. has been associated with HPV infection, persistent Hr-HPV infection, and cervical lesions compared to control groups [58–60]. Moreover, *Prevotella *spp. have been associated with persistent CIN lesions and slower regression of disease [52]. Despite limited reports on *P. amnii*, it was the most abundant bacteria in women with low-grade SILs in a Chinese study cohort [60].

Abnormal cytology or dysplasia, such as abnormal Pap smears, SILs, cervical dysplasia, and CIN in Latinas, yielded associations with 24 unique bacteria. Across multiple studies in this category, *C. trachomatis* was significantly enriched, and seven additional bacteria were consistently enriched although not significant: *Fannyhessea *spp., *G. vaginalis, L. crispatus, L. iners, Prevotella* spp., *P. amnii*, and *Sneathia* spp.. In our review, some conditions included depleted bacteria, while most reported enriched bacteria. For example, *L. iners* and *L. crispatus* were depleted in women with SILs, and *Fannyhessea *spp. were depleted in women with CIN. An enrichment of *G. vaginalis* was observed across three conditions: abnormal Pap smears, cervical dysplasia, and CIN. An abundance of *G. vaginalis* has been frequently reported in the vaginal microbiome of women with HPV and Hr-HPV infection and is also associated with HPV persistence [61, 62]. A study revealed that coinfections with HPV and *G. vaginalis* increased risk for SILs and cervical cancer [61]. This may be due to the ability of *G. vaginalis* and other BV-associated bacteria to secrete sialidase, which is an enzyme that has been linked to an increased risk of cervical lesions [63]. In our review, *L. iners, L. crispatus*, and *Fannyhessea *spp. had conflicting relative abundance reports in women with cervical dysplasia. Existing literature suggests that *Fannyhessea *spp. are frequently detected in women with high-grade CIN [64, 65] and cervical cancer [64–66] in Chinese, South Korean, and Slovak cohorts.

Our literature review revealed that cervical cancer includes the enrichment of four bacteria and the depletion of one in Latinas. The depleted bacterium in Latinas with cervical cancer was *L. crispatus*, which is generally associated with a healthy cervicovaginal microbiome [46]. *Fusobacterium * and *Sneathia *spp. were enriched across three studies in our review on cervical cancer in Latinas. *Fusobacterium *spp. were enriched in Latinas with HPV/Hr-HPV infection and cervical cancer. Existing evidence demonstrates that *Fusobacterium *spp. abundance is strongly associated with HPV infection and high-grade dysplasia [22, 52, 58]. Moreover, after examining 112 cervical cancer tumor tissues, Huang et al. (2020) suggest *Fusobacterium *spp. may be a potential diagnostic and prognostic biomarker for cervical cancer [67]. Lastly, *Sneathia *spp. were enriched across HPV/Hr-HPV infection, abnormal cytology/dysplasia, and cervical cancer in Latinas in two included articles. One study considered *Sneathia *spp. a microbiological marker of Hr-HPV infection, given that *Sneathia *spp. were detected three times more frequently in women with Hr-HPV infection [52]. Moreover, previous studies on non-Latinas reported a significantly greater abundance of *Sneathia *spp. in women with cervical intraepithelial lesions, high-grade SILs, and cervical cancer compared to healthy controls [68, 69]. Furthermore, our lab demonstrated that *Sneathia* spp. exhibit potential oncogenic mechanisms based on the altered immunometabolic microenvironment in a 3D model of the human cervix [70, 71]. Although we were unable to run a meta-analysis, *Sneathia* spp. are microbes of interest and require further investigation in Latina cohorts and longitudinal studies  considering they are  enriched in all stages of cervical carcinogenesis.

Other significant findings related to alpha and beta diversity in Latinas were also reported. Two studies in our review reported higher species richness and diversity in Latinas with high-grade cervical intraepithelial neoplasia (CIN3) compared to low-grade cervical intraepithelial neoplasia (CIN1) and Latinas with SILs compared to non-SILs. Existing literature reports higher alpha diversity among women with abnormal cervical pathology, with a trend to increase the more severe the cervical lesion [72]. In addition, a previous study reported higher alpha diversity among women with cervical cancer, which was confirmed in our review on Latinas [73]. Our review also revealed a significantly higher beta diversity in women with SILs compared to non-SILs and women with cervical cancer compared to those with HPV infection and cervical dysplasia. Significant differences in beta diversity among non-Latina women with HPV infection, SILs, and cervical cancer compared to controls were also reported in a systematic review [74]. Overall, our review revealed a trend toward increased alpha and beta diversity and cervical carcinogenesis progression in Latinas.

Regarding *Lactobacillus* dominance, two studies in our review reported *L. iners*, a transitional bacterium, as the most abundant among Latinas [47]. Lastly, two studies in our review revealed significant depletion of *Lactobacillus* species among Latinas compared to non-Latinas. Previously, Ravel et al. (2011) demonstrated a racial–ethnic difference in microbiome composition as  Black and Latina women harbor increased levels of diverse anaerobes and lower levels of health-associated, *Lactobacillus* species compared to White and Asian women who possess *Lactobacillus*-dominant vaginal microbiomes [75, 76]. In our review, we observed similar overall low levels of *Lactobacillus* and increased bacterial diversity across Latinas globally.

Our review featured studies from ten countries across North, Central, and South America and the Caribbean. Another review of women from Latin America and the Caribbean (LAC) revealed that early age at first sexual intercourse, number of sexual partners, and sexual behavior of the partner are associated with increased risk of genital HPV acquisition [77]. Additional co-factors for cervical cancer in LAC include high parity, long-term use of oral contraceptives, high prevalence of smoking, and co-infection with the human immunodeficiency virus (HIV) [77]. In addition to these risk factors, women in LAC face systematic barriers to healthcare such as uneven resource distribution, variable infrastructure, and healthcare service availability [77, 78]. Rural, low-resourced, and underserved populations in LAC are especially impacted and are less likely to have access to HPV vaccination, cervical cancer prevention, and screening [52]. Studies along the U.S.–Mexican border also demonstrate the need for increased health education and awareness regarding the HPV vaccine and HPV/cervical disease diagnoses to prevent cervical cancer with screening, early diagnosis, and treatment [9, 79].

### Strengths and Limitations

To our knowledge, this is the first systematic review to report on the cervicovaginal bacteria in studies related to HPV infection, cervical dysplasia, and cancer in Latinas. The strengths of this review include strict criteria for inclusion, yielding only articles relevant to our research question. While our inclusion criteria were strict, our search generated a broad range of articles focused on our population of interest. By including the extensive Latinx search hedge, we were able to account for the racial–ethnic, geographic, and linguistic range the individual “Hispanic” search term lacks [27].

The limitations are those inherent to a systematic review, which includes relying on the information and quality of data available. Consequently, the varying study designs and general lack of homogeneity of reported analyses (e.g., few and dissimilar statistical tests, differing variables, etc.) failed to meet the basic criteria for unbiased meta-analytic methods. In an effort to update the review and reassess the potential for a meta-analysis, the search could be re-conducted after a few years following publication of additional studies. With more data available, significant associations of specific bacteria can be determined in Latinas with HPV, cervical dysplasia, and cervical cancer. Second, some studies adjusted for confounding factors and excluded individuals with STIs (i.e., *C. trachomatis*), while other studies included all patients, even those with STIs. This leads to a discrepancy in the inclusion or exclusion of individuals with *C. trachomatis* in the 25 included studies. As such, the *C. trachomatis* findings must be interpreted with caution. Hence, controlling for confounding variables involves balancing the need to include all individuals with the demand of strict bias criteria. Lastly, in order to establish a causal connection between the enriched bacteria and cervical carcinogenesis, longitudinal microbiome studies must be performed [80]. Longitudinal microbiome studies and larger cohort studies, including Latinas will help determine the role of bacteria as drivers (influential disease-causing agents), passengers (less influential agents favoring the environment), or a consequence of disease in this population of women [80].

### Conclusions and Implications

The systematic review identified 42 unique bacteria across 25 studies related to HPV infection, cervical dysplasia, and cervical cancer in Latinas. *L. crispatus* was enriched in healthy Latinas and those with abnormal cytology/dysplasia, while *L. iners* was enriched in multiple states ranging from healthy to abnormal cytology/dysplasia groupings. *C. trachomatis, P. amnii,* and *Prevotella *spp. were enriched in Latinas with HPV/Hr-HPV infection and abnormal cytology/dysplasia. Notably, *Fusobacterium* spp. were enriched in Latinas with HPV/Hr-HPV infection and cervical cancer. Importantly, *Sneathia *spp. were enriched across all stages in cervical cancer progression—HPV/Hr-HPV infection, abnormal cytology/dysplasia, and cervical cancer. In addition to more research on Latina populations, barriers related to social determinants of health and structural racism must be considered to improve outcomes against a preventable disease like cervical cancer amongst Latinas. Advanced public health efforts, including community-based participatory research projects with Latinas, can reduce health disparities in HPV infection and cervical cancer [81, 82]. In conclusion, future epidemiological studies must intentionally include Latina women in order to ultimately create primary or secondary preventative strategies for this susceptible population.

### Supplementary Information

Below is the link to the electronic supplementary material.

Supplementary file1 (DOCX 20 KB)

Supplementary file2 (XLSX 52 KB)

## Data Availability

All manuscript data are included as electronic supplementary material.

## Abbreviations

HPV: Human papillomavirus
Hr-HPV: High-risk human papillomavirus
PRISMA: Preferred Reporting Items for Systematic Reviews and Meta-analyses
LAC: Latin America and the Caribbean
CIN: Cervical intraepithelial neoplasia
CIN1: Cervical intraepithelial neoplasia, low grade
CIN3: Cervical intraepithelial neoplasia, high grade
BV: Bacterial vaginosis
STI: Sexually transmitted infection
ROBINS-E: Risk Of Bias In Non-randomized Studies of Exposure
*L.**iners*: *Lactobacillus* *iners*
*C.**trachomatis*: *Chlamydia trachomatis*
*L.**crispatus*: *Lactobacillus* *crispatus*
*G.**vaginalis*: *Gardnerella* *vaginalis*
Spp.: Species
*P.**amnii*: *Prevotella* *amnii*
SILs: Squamous intraepithelial lesions
Pap smear: Papanicolaou smear
HIV: Human immunodeficiency virus
U.S: United States
NParticipants: Number of total participants
NOutcomeGrp: Number of participants in the outcome group
NCtrlGrp: Number of participants in the control group
CC: Cervical cancer
