# Supplementary material for: The Vaginal Microbiota, Human Papillomavirus Infection, and Cervical Carcinogenesis: A Systematic Review in the Latina Population
Source: J Epidemiol Glob Health. 2024 Feb 26;14(2):480–97. doi: 10.1007/s44197-024-00201-z (PMC11176136; doi:10.1007/s44197-024-00201-z)
Supplement: Supplementary file 1 — Supplementary file1 (DOCX 20 KB) [file 44197_2024_201_MOESM1_ESM.docx]

1. Search strategies – performed on 11/9/2022

1.1 PubMed

| Search | Hits |
| --- | --- |
| ("Microbiota"[Mesh] |  |
| AND Vaginal OR "Cervix Uteri"[Mesh] |  |
| AND ((hispanic*[tiab] OR "hispanic american*"[tiab] OR "hispano*"[tiab] OR "latine*"[tiab] OR "latina*"[tiab] OR "latin"[tiab] OR "latinu*"[tiab] OR "latino"[tiab] OR "latinx*"[tiab] OR "latin american*"[tiab] OR "latin america"[Mesh] OR "spanish speak*"[tiab] OR "mexico*"[tiab] OR "Mexico"[Mesh] OR "cuban*"[tiab] OR "peruvian*"[tiab] OR "dominican*"[tiab] OR "brazilian*"[tiab] OR "central american*"[tiab] OR "costa rican*"[tiab] OR "guatemalan*"[tiab] OR "honduran*"[tiab] OR "uruguayan*"[tiab] OR "argentina"[Mesh] OR "argentine"[tiab] OR "argentinian"[tiab] OR "argentinean"[tiab] OR "panamanian*"[tiab] OR "salvadorean*"[tiab] OR "salvadoran*"[tiab] OR "salvadorian*"[tiab] OR "nicaraguan*"[tiab] OR "south america*"[tiab] OR "bolivian*"[tiab] OR "chilean*"[tiab] OR "Chile"[Mesh] OR "colombian*"[tiab] OR "ecuadorian*"[tiab] OR "paraguay*"[tiab] OR "Paraguay"[Mesh] OR "venezuelan*"[tiab] OR "puerto rican*"[tiab] OR "puerto rico*"[tiab] OR "Puerto Rico"[Mesh] OR "spanish america*"[tiab] OR "boricua*"[tiab] OR "chicana*"[tiab] OR "chicano"[tiab] OR "Hispanic Americans"[Mesh] OR "latinoamerican*"[tiab] OR "Mexican Americans"[Mesh] OR "spanish caribbean*"[tiab] OR "mexican american*"[tiab]) AND ("united states"[MeSH] OR "united state*"[tiab] OR "north america*"[tiab] OR "north america"[MeSH] OR "Midwestern United States"[Mesh] OR "Southeastern United States"[Mesh] OR "Southwestern United States"[Mesh] OR "Northwestern United States"[Mesh] OR "Appalachia*"[tiab] OR "great lakes"[tiab] OR "mid atlantic state*"[tiab] OR "mid atlantic region*"[tiab] OR "middle atlantic state*"[tiab] OR "middle atlantic region*"[tiab] OR "midwestern us*"[tiab] OR "midwestern state*"[tiab] OR "midwest state*"[tiab] OR "midwest us*"[tiab] OR "great plains"[tiab] OR "heartland"[tiab] OR "new england"[tiab] OR "northeastern us*"[tiab] OR "northeastern state*"[tiab] OR "northeast state*"[tiab] OR "northeast us*"[tiab] OR "pacific northwest"[tiab] OR "northwestern us*"[tiab] OR "northwest us*"[tiab] OR "northwestern state*"[tiab] OR "northwest state*"[tiab] OR "pacific state*"[tiab] OR "southeast state*"[tiab] OR "southeastern state*"[tiab] OR "southeast region"[tiab] OR "southeastern region"[tiab] OR "southeast us*"[tiab] OR "southeastern us*"[tiab] OR "southern state*"[tiab] OR "southern us*"[tiab] OR "southwest state*"[tiab] OR "southwestern state*"[tiab] OR "southwest us*"[tiab] OR "southwestern us*"[tiab] OR "deep south"[tiab] OR "black belt"[tiab] OR "rust belt"[tiab] OR "district of Columbia"[tiab] OR "Washington dc"[tiab] OR "Alabama"[tiab] OR "Alaska"[tiab] OR "Arizona"[tiab] OR "Arkansas"[tiab] OR "California"[tiab] OR "Colorado"[tiab] OR "Connecticut"[tiab] OR "Delaware"[tiab] OR "Florida"[tiab] OR "Georgia"[tiab] OR "Hawaii"[tiab] OR "Hawai i"[tiab] OR "Idaho"[tiab] OR "Illinois"[tiab] OR "Indiana"[tiab] OR "Iowa"[tiab] OR "Kansas"[tiab] OR "Kentucky"[tiab] OR "Louisiana"[tiab] OR "Maine"[tiab] OR "Maryland"[tiab] OR "Massachusetts"[tiab] OR "Michigan"[tiab] OR "Minnesota"[tiab] OR "Minneapolis"[tiab] OR "Mississippi"[tiab] OR "Missouri"[tiab] OR "Montana"[tiab] OR "Nebraska"[tiab] OR "Nevada"[tiab] OR "New Hampshire"[tiab] OR "New Jersey"[tiab] OR "New Mexico"[tiab] OR "New York"[tiab] OR "North Carolina"[tiab] OR "North Dakota"[tiab] OR "Ohio"[tiab] OR "Oklahoma"[tiab] OR "Oregon"[tiab] OR "Pennsylvania"[tiab] OR "Rhode Island"[tiab] OR "South Carolina"[tiab] OR "South Dakota"[tiab] OR "Tennessee"[tiab] OR "Texas"[tiab] OR "Utah"[tiab] OR "Vermont"[tiab] OR "Virginia"[tiab] OR "Washington"[tiab] OR "West Virginia"[tiab] OR "Wisconsin"[tiab] OR "Wyoming"[tiab] OR america*[tiab] OR "Americas"[Mesh]) |  |
| Filters: Female |  |
| Filters: from 2000 - 2022 |  |
|  | 178 |

1.2 EMBASE

| Search | Hits |
| --- | --- |
| ('microbiota'/exp OR microbiota) |  |
| AND (vaginal OR 'cervix'/exp OR cervix) |  |
| AND (hispanic*:ti,ab OR hispano*:ti,ab OR latine*:ti,ab OR latina*:ti,ab OR latin:ti,ab OR latinu*:ti,ab OR latino:ti,ab OR latinx*:ti,ab OR 'latin american*':ti,ab OR 'south and central america'/exp OR 'south and central america' OR 'spanish speak*':ti,ab OR mexico*:ti,ab OR 'mexico'/exp OR 'mexico' OR cuban*:ti,ab OR peruvian*:ti,ab OR dominican*:ti,ab OR brazilian*:ti,ab OR 'central american*':ti,ab OR 'costa rican*':ti,ab OR guatemalan*:ti,ab OR honduran*:ti,ab OR uruguayan*:ti,ab OR argentine:ti,ab OR argentinian:ti,ab OR argentinean:ti,ab OR panamanian*:ti,ab OR salvadoran*:ti,ab OR salvadorean*:ti,ab OR salvadorian*:ti,ab OR nicaraguan*:ti,ab OR 'south america*':ti,ab OR bolivian*:ti,ab OR chilean*:ti,ab OR colombian*:ti,ab OR ecuadorian*:ti,ab OR paraguay*:ti,ab OR venezuelan*:ti,ab OR 'puerto rican*':ti,ab OR 'puerto rico*':ti,ab OR 'puerto rico'/exp OR 'puerto rico' OR 'dominican republic'/exp OR 'dominican republic' OR 'cuba'/exp OR 'cuba' OR 'spanish america*':ti,ab OR boricua*:ti,ab OR chicana*:ti,ab OR chicano:ti,ab OR hispano:ti,ab OR 'hispanic american*':ti,ab OR 'hispanic'/exp OR 'hispanic' OR latinoamerican*:ti,ab OR 'mexican american'/exp OR 'mexican american' OR 'spanish caribbean*':ti,ab OR 'mexican american*':ti,ab) AND ('united states'/exp OR 'united states' OR 'united state*':ti,ab OR 'north america*':ti,ab OR 'north america'/exp OR 'north america' OR appalachia*:ti,ab OR 'great lakes':ti,ab OR 'mid atlantic state*':ti,ab OR 'mid atlantic region*':ti,ab OR 'middle atlantic state*':ti,ab OR 'middle atlantic region*':ti,ab OR 'midwestern us*':ti,ab OR 'midwestern state*':ti,ab OR 'midwest state*':ti,ab OR 'midwest us*':ti,ab OR 'great plains':ti,ab OR heartland:ti,ab OR 'new england':ti,ab OR 'northeastern us*':ti,ab OR 'northeastern state*':ti,ab OR 'northeast state*':ti,ab OR 'northeast us*':ti,ab OR 'pacific northwest':ti,ab OR 'northwestern us*':ti,ab OR 'northwest us*':ti,ab OR 'northwestern state*':ti,ab OR 'northwest state*':ti,ab OR 'pacific state*':ti,ab OR 'southeast state*':ti,ab OR 'southeastern state*':ti,ab OR 'southeast region':ti,ab OR 'southeastern region':ti,ab OR 'southeast us*':ti,ab OR 'southeastern us*':ti,ab OR 'southern state*':ti,ab OR 'southern us*':ti,ab OR 'southwest state*':ti,ab OR 'southwestern state*':ti,ab OR 'southwest us*':ti,ab OR 'southwestern us*':ti,ab OR 'deep south':ti,ab OR 'black belt':ti,ab OR 'rust belt':ti,ab OR 'district of columbia':ti,ab OR 'washington dc':ti,ab OR alabama:ti,ab OR alaska:ti,ab OR arizona:ti,ab OR arkansas:ti,ab OR california:ti,ab OR colorado:ti,ab OR connecticut:ti,ab OR delaware:ti,ab OR florida:ti,ab OR georgia:ti,ab OR hawaii:ti,ab OR 'hawai i':ti,ab OR idaho:ti,ab OR illinois:ti,ab OR indiana:ti,ab OR iowa:ti,ab OR kansas:ti,ab OR kentucky:ti,ab OR louisiana:ti,ab OR maine:ti,ab OR maryland:ti,ab OR massachusetts:ti,ab OR michigan:ti,ab OR minnesota:ti,ab OR minneapolis:ti,ab OR mississippi:ti,ab OR missouri:ti,ab OR montana:ti,ab OR nebraska:ti,ab OR nevada:ti,ab OR 'new hampshire':ti,ab OR 'new jersey':ti,ab OR 'new mexico':ti,ab OR 'new york':ti,ab OR 'north carolina':ti,ab OR 'north dakota':ti,ab OR ohio:ti,ab OR oklahoma:ti,ab OR oregon:ti,ab OR pennsylvania:ti,ab OR 'rhode island':ti,ab OR 'south carolina':ti,ab OR 'south dakota':ti,ab OR tennessee:ti,ab OR texas:ti,ab OR utah:ti,ab OR vermont:ti,ab OR virginia:ti,ab OR washington:ti,ab OR 'west virginia':ti,ab OR wisconsin:ti,ab OR wyoming:ti,ab OR america*:ti,ab OR 'western hemisphere'/exp OR 'western hemisphere') |  |
| AND [female] |  |
| lim AND [2000-2022]/py |  |
|  | 168 |

1.3 Scopus

| Search | Hits |
| --- | --- |
| ( ( TITLE-ABS-KEY ( microbiota ) |  |
| AND TITLE-ABS-KEY ( vagina OR cervix ) ) ) |  |
| AND ( ( INDEXTERMS ( ( "Hispanic Americans" ) OR ( "Mexican Americans" ) OR ( "latin america" ) OR ( "Mexico" ) OR ( "argentina" ) OR ( "Chile" ) OR ( "Paraguay" ) OR ( "Puerto Rico" ) OR ( "Americas" ) OR ( "Western Hemisphere" ) ) OR ( TITLE-ABS-KEY ( ( "hispanic*" ) OR ( "hispanic american*" ) OR ( "hispano*" ) OR ( "latine*" ) OR ( "latina*" ) OR ( "latin" ) OR ( "latinu*" ) OR ( "latino" ) OR ( "latinx*" ) OR ( "latin american*" ) OR ( "spanish speak*" ) OR ( "mexico*" ) OR ( "cuban*" ) OR ( "peruvian*" ) OR ( "dominican*" ) OR ( "brazilian*" ) OR ( "central american*" ) OR ( "costa rican*" ) OR ( "guatemalan*" ) OR ( "honduran*" ) OR ( "uruguayan*" ) OR ( "argentine" ) OR ( "argentinian" ) OR ( "argentinean" ) OR ( "panamanian*" ) OR ( "salvadoran*" ) OR ( "salvadorean*" ) OR ( "salvadorian" ) OR ( "nicaraguan*" ) OR ( "south america*" ) OR ( "bolivian*" ) OR ( "chilean*" ) OR ( "colombian*" ) OR ( "ecuadorian*" ) OR ( "paraguayan*" ) OR ( "venezuelan*" ) OR ( "puerto rican*" ) OR ( "puerto rico*" ) OR ( "spanish america*" ) OR ( "boricua*" ) OR ( "chicana*" ) OR ( "chicano*" ) OR ( "latinoamerican*" ) OR ( "spanish caribbean*" ) OR ( "mexican american*" ) ) ) AND ( INDEXTERMS ( ( "united states" ) OR ( "north america" ) OR ( "Midwestern United States" ) OR ( "Southeastern United States" ) OR ( "Southwestern United States" ) OR ( "Northwestern United States" ) ) ) OR ( TITLE-ABS-KEY ( ( "united state*" ) OR ( "america*" ) OR ( "north america*" ) OR ( "Appalachia*" ) OR ( "great lakes" ) OR ( "mid atlantic state*" ) OR ( "mid atlantic region*" ) OR ( "middle atlantic state*" ) OR ( "middle atlantic region*" ) OR ( "midwestern us*" ) OR ( "midwestern state*" ) OR ( "midwest state*" ) OR ( "midwest us*" ) OR ( "great plains" ) OR ( "heartland" ) OR ( "new england" ) OR ( "northeastern us*" ) OR ( "northeastern state*" ) OR ( "northeast state*" ) OR ( "northeast us*" ) OR ( "pacific northwest" ) OR ( "northwestern us*" ) OR ( "northwest us*" ) OR ( "northwestern state*" ) OR ( "northwest state*" ) OR ( "pacific state*" ) OR ( "southeast state*" ) OR ( "southeastern state*" ) OR ( "southeast region" ) OR ( "southeastern region" ) OR ( "southeast us*" ) OR ( "southeastern us*" ) OR ( "southern state*" ) OR ( "southern us*" ) OR ( "southwest state*" ) OR ( "southwestern state*" ) OR ( "southwest us*" ) OR ( "southwestern us*" ) OR ( "deep south" ) OR ( "black belt" ) OR ( "rust belt" ) OR ( "district of Columbia" ) OR ( "Washington dc" ) OR ( "Alabama" ) OR ( "Alaska" ) OR ( "Arizona" ) OR ( "Arkansas" ) OR ( "California" ) OR ( "Colorado" ) OR ( "Connecticut" ) OR ( "Delaware" ) OR ( "Florida" ) OR ( "Georgia" ) OR ( "Hawaii" ) OR ( "Hawai i" ) OR ( "Idaho" ) OR ( "Illinois" ) OR ( "Indiana" ) OR ( "Iowa" ) OR ( "Kansas" ) OR ( "Kentucky" ) OR ( "Louisiana" ) OR ( "Maine" ) OR ( "Maryland" ) OR ( "Massachusetts" ) OR ( "Michigan" ) OR ( "Minnesota" ) OR ( "Minneapolis" ) OR ( "Mississippi" ) OR ( "Missouri" ) OR ( "Montana" ) OR ( "Nebraska" ) OR ( "Nevada" ) OR ( "New Hampshire" ) OR ( "New Jersey" ) OR ( "New Mexico" ) OR ( "New York" ) OR ( "North Carolina" ) OR ( "North Dakota" ) OR ( "Ohio" ) OR ( "Oklahoma" ) OR ( "Oregon" ) OR ( "Pennsylvania" ) OR ( "Rhode Island" ) OR ( "South Carolina" ) OR ( "South Dakota" ) OR ( "Tennessee" ) OR ( "Texas" ) OR ( "Utah" ) OR ( "Vermont" ) OR ( "Virginia" ) OR ( "Washington" ) OR ( "West Virginia" ) OR ( "Wisconsin" ) OR ( "Wyoming" ) ) ) ) ) |  |
|  | 19 |

The vaginal microbiota, HPV, and cervical dysplasia: a systematic review in the Latina population. Journal of Epidemiology and Global Health. Vianney Mancilla, BA^1^; Nicole R. Jimenez, PhD^2^; Naomi Bishop, MLIS^1^; Melissa Flores, PhD^3^, and Melissa M. Herbst-Kralovetz, PhD^1,2^. ^1^Department of Basic Medical Sciences, University of Arizona College of Medicine - Phoenix, Phoenix, AZ, ^2^Department of Obstetrics and Gynecology, University of Arizona College of Medicine - Phoenix, Phoenix, AZ, ^3^Department of Psychology, University of Arizona, Tucson, AZ. [mherbst1@arizona.edu](mailto:mherbst1@arizona.edu)
